# The Opposite Effect of Metal Ions on Short-/Long-Range Water Structure: A Multiple Characterization Study

**DOI:** 10.3390/ijms17050602

**Published:** 2016-04-25

**Authors:** Kai Ma, Lin Zhao

**Affiliations:** School of Environmental Science and Engineering, Tianjin University, Tianjin 300072, China; makai@tju.edu.cn

**Keywords:** metal ion, spectrometer, molecular dynamics simulations, long-range effect

## Abstract

Inorganic electrolyte solutions are very important in our society as they dominate many biochemical and geochemical processes. Herein, an in-depth study was performed to illustrate the ion-induced effect on water structure by coupling NMR, viscometer, Raman and Molecular Dynamic (MD) simulations. The NMR coefficient (*B*_NMR_) and diffusion coefficient (*D*) from NMR, and viscosity coefficient (*B*_vis_) from a viscometer all proved that dissolved metal ions are capable of enhancing the association degree of adjacent water molecules, and the impact on water structure decreased in the order of Cr^3+^ > Fe^3+^ > Cu^2+^ > Zn^2+^. This regularity was further evidenced by Raman analysis; however, the deconvoluted Raman spectrum indicated the decrease in high association water with salt concentration and the increase in low association water before 200 mmol·L^−1^. By virtue of MD simulations, the opposite changing manner proved to be the result of the opposite effect on short-/long-range water structure induced by metal ions. Our results may help to explain specific protein denaturation induced by metal ions.

## 1. Introduction

It is well known that salt solution, as compared to pure water, is characterized by four colligative properties, *i.e.*, freezing point depression, boiling point elevation, vapor pressure lowering, and osmotic pressure [1], which makes it used in dialysis, melting snow, *etc.* Besides anions, cations dissolved in aqueous solution have proved to be the key to the changes in water properties [1,2]. In addition, metal ions can also interact with a variety of biomolecules, such as protein [3] and DNA [4], and further lead to their structural changes to different extents. In this process, metal ions may bind directly with the functional groups on biomolecules [3,5] but may also indirectly force biomolecules to degenerate by alternating the water structure surrounding the biomolecules [6]. As a result, a complete knowledge of solution structure is very important to understand metal ion-related chemical and biological phenomena occurring in our daily life.

In recent years, the effects of dissolved metal ions on surrounding water molecules have been extensively characterized by Terahertz Time-Domain Spectroscopy (THz TDS), Raman/IR, NMR, Molecular Dynamic (MD) simulations, *etc.* Kropman and Bakker [7] found the hydrogen-bonding (HB) dynamics of water molecules solvating Cl^−^, Br^−^, I^−^ anions slow compared with pure water, indicating the remarkable influence of center ions on solvating water molecules. Furthermore, this group observed significant dependence of vibrational lifetime of hydration water on the nature of metal ions [8], but the enhancement of the HB network upon adding ions is confined within the first solvation shell as indicated by the changes in rotational dynamics [9]. In addition to HB dynamics, static water structures have also been determined to study the metal ion-induced effects. An X-ray diffraction study suggested that the KCl unit in aqueous solution could control 45 water molecules to form rigid spherical structure [10]. Another neutron diffraction study provided compelling evidence that Ca^2+^ could have an impact on the hydrogen structure in the second hydration shell [11]. In 2012, an IR photodissociation study on water nanodrops involving ions, manifested that the ion-induced effect on water structure propagates all the way to the distance more than 1 nm from the ion, corresponding to 250 water molecules [12]. Thus, ions are very likely to have effects on both the adjacent hydration water and those far away from center ions, which is still waiting for further study.

Moreover, the ion-induced effects are always ion-specific. For example, K^+^ and Cs^+^ were found to have “negative hydration” effect, which means that the water molecules near the ions become more mobile than in pure water [13]. On the contrary, the bivalent ion, Mg^2+^, was found to interact intensively with molecules in the first solvation shell [14], and to enhance the HB intensity within aqueous solution [15]. As for another monovalent, Na^+^ just manifested a very slight effect on the average diffusion of water [16], and has been believed to be the borderline between strong and weak interaction [17]. This difference may be the origin of “kosmotropes” and “chaotropes”. Even though still controversial, this classification has suggested that the stark contrast in ion-related effects may originate from ionic charge [15], ionic radius [18], or ionic charge density [18,19].

Interestingly, a previous THz TDS study showed that, the dissolved cations can have an effect on both hydration water molecules and those beyond hydration shells [20]. To further address this suspending problem, multiple characterization methods were adopted to analyze the structural features of CrCl_3_, FeCl_3_, CuCl_2_ and ZnCl_2_ solutions. In this work, NMR coefficient (*B*_NMR_) and diffusion coefficient (*D*) were obtained to analyze the ion-induced influence on water association degree and to evaluate the difference among the four metal ions. Then, the alteration in water structure nearby was further discussed by comparing viscosity coefficient (*B*_vis_). Subsequently, the Raman spectra of aqueous solutions with different concentrations were recorded and deconvoluted to find out the changes in content of different water species upon ion addition. Finally, the ion-induced effect on short-/long-range water structure was confirmed by studying Radial Distribution Function (RDF) obtained from MD simulations.

## 2. Results

### 2.1. NMR Analysis

NMR is very sensitive to the changes in chemical environment of specific element, which makes it suitable for solution structure characterization. Here, we make use of two characteristic parameters from NMR to analyze the variations in association degree of hydrogen bond network upon adding different electrolytes into the solutions.

#### 2.1.1. Spin-Lattice Relaxation Time (*T*_1_)

**Figure 1:**
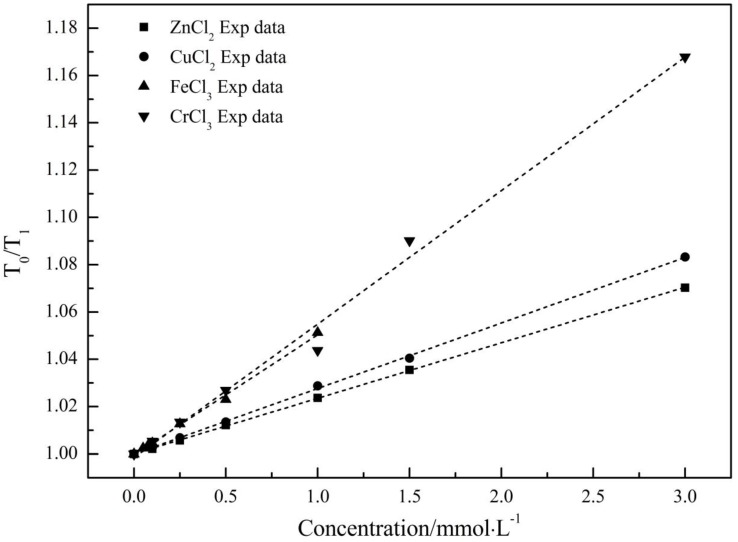
*T*_0_/*T*_1_ of ZnCl_2_, CuCl_2_, FeCl_3_ and CrCl_3_ aqueous solutions at different concentrations. *T*_0_ is the spin-lattice relaxation time of pure water. Short dashed lines are the linear fitting results corresponding to the experimental data measured by NMR.

*T*_1_ can reflect the process of energy exchange between excited atoms and surroundings, in other words, the smaller *T*_1_ means the stronger bounds between target atoms and surroundings within the system. *T*_0_ is the spin-lattice relaxation time of pure water. The *T*_1_ of ZnCl_2_, CuCl_2_, FeCl_3_ and CrCl_3_ aqueous solutions at different concentrations were shown Figure 1. From Figure 1, we can see that the *T*_0_/*T*_1_ of the four electrolyte solutions increased with concentrations in a linear fashion, which is in accordance with the observations from Engel and Hertz [21].

According to Engel and Hertz [21], the relation between *T*_0_, *T*_1_ and solution concentration could be depicted by Equation (1):

(1) $$\left[\left(1/T_{1}\right)/\left(1/T_{0}\right)\right]=1+B_{NMR}C+\cdot \cdot \cdot$$

where *T*_0_, *T*_1_ are the spin-lattice relaxation time of pure water and aqueous solution, respectively, ms. *C* is the solution concentration, mmol·L^−1^. *B*_NMR_ is the NMR coefficient, L·mmol^−1^, and could reflect the influence of electrolyte on the association degree of the solution system. Hence, four empirical Equations (2)–(5) were obtained by linear fitting the experimental data to Equation (1), and the fitting line can be seen in Figure 1.

(2) $$T_{0}/T_{1}=0.99844+0.05646C_{{\text{CrCl}}_{3}}\;R^{2}=0.99023$$

(3) $$T_{0}/T_{1}=0.99959+0.05082C_{{\text{FeCl}}_{3}}\;R^{2}=0.99681$$

(4) $$T_{0}/T_{1}=0.99996+0.02771C_{{\text{CuCl}}_{2}}\;R^{2}=0.99943$$

(5) $$T_{0}/T_{1}=1.00005+0.02349C_{{\text{ZnCl}}_{2}}\;R^{2}=0.99986$$

According to the four equations, the *B*_NMR_ were all larger than zero, which means the association degree of the aqueous solutions on average are higher than that of pure water due to the addition of four electrolytes. Importantly, it has been reported that Cl^−^ has negligible effect on water hydrogen bond network [22,23], so the difference in association degree between aqueous solution and pure water is due mainly to the metal ions appearing in the solution. Considering that there are no other additives except for metal chloride in all sample solutions, the discussion on structural changes in the following parts would just be proceeded based on the differences in dissolved metal ions.

#### 2.1.2. Diffusion Coefficient (*D*)

The molecular translational motion in solution is commonly referred to as self-diffusion and is defined with a self-diffusion coefficient *D* [24]. According to Cohen *et al.* [25], compared with free diffusion, the restricted diffusion was characterized by a modified diffusion behavior as indicated by the mean displacement experienced by the diffusing molecular species. Thus, the force on translational water molecule exerted by metal ions could be manifested by *D*.

**Figure 2:**
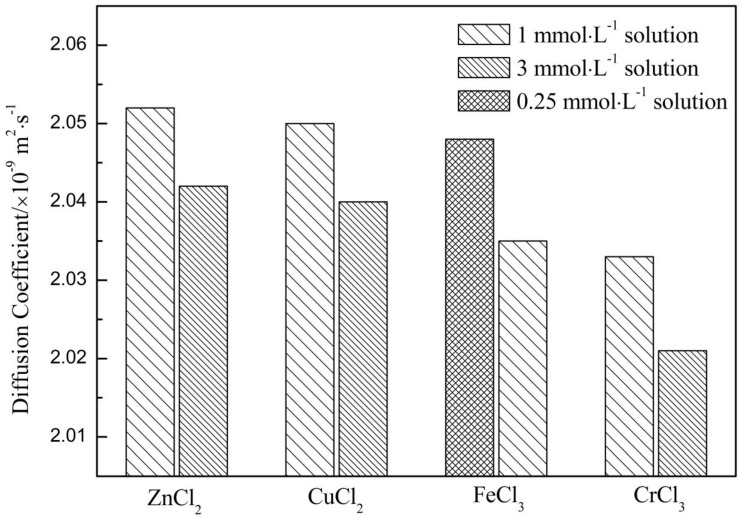
Comparison of diffusion coefficient of four electrolyte aqueous solutions at different concentrations. The solutions with identical concentration are designated as the same fill pattern of the histogram.

First, we obtained a diffusion coefficient of H_2_O at 298.15 K, *D* = 2.058 × 10^−9^ m^2^·s^−1^, which was almost identical to another Pulsed Gradient Spin-Echo (PGSE) NMR result (2.11 ± 0.20 × 10^−9^ m^2^·s^−1^) acquired under the same condition [26]. However, the diffusion coefficient of D_2_O (1.762 × 10^−9^ m^2^·s^−1^) obtained here was much smaller than that of H_2_O. Up to now, many IR studies have shown that D_2_O bears hydrogen bond interaction stronger than H_2_O at the presence of the red-shift of OH stretching vibration peak [27,28,29]. Thus, the difference in *D* just reflects the enhancement in hydrogen bond network of D_2_O. Furthermore, the diffusion coefficient of four electrolyte aqueous solutions could be compared in Figure 2. It is obvious that the diffusion coefficients of aqueous solutions are smaller than that of H_2_O but much larger than that of D_2_O at the meantime. In addition, the diffusion coefficients of the aqueous solutions would further decrease with metal ion concentration increasing from 1 to 3 mmol·L^−1^ for ZnCl_2_, CuCl_2_ and CrCl_3_ solutions, from 0.25 to 1 mmol·L^−1^ for FeCl_3_ solutions. That is to say, the translational motion of water molecule on average would be further confined with increasing salt concentration.

Moreover, according to Figure 2, the diffusion coefficients of 1 mmol·L^−1^ metal ion solution followed the order: *D*(H_2_O) > *D*(ZnCl_2_) > *D*(CuCl_2_) > *D*(FeCl_3_) > *D*(CrCl_3_) > *D*(D_2_O). That is to say, the ability in enhancing association degree of water decrease in the order of Cr^3+^ > Fe^3+^ > Cu^2+^ > Zn^2+^. Not only that, the *B*_NMR_ for the four electrolytes also followed the order: Cr^3+^ > Fe^3+^ > Cu^2+^ > Zn^2+^ (see Section 2.1.1). The identical order of *D* and *B*_NMR_ indicated that the NMR is suitable for manifesting the metal ion-induced effect on water structure. The concrete reason for the difference in changing water structure would be discussed by combining viscosity and Raman results.

### 2.2. Viscosity Measurement

**Figure 3:**
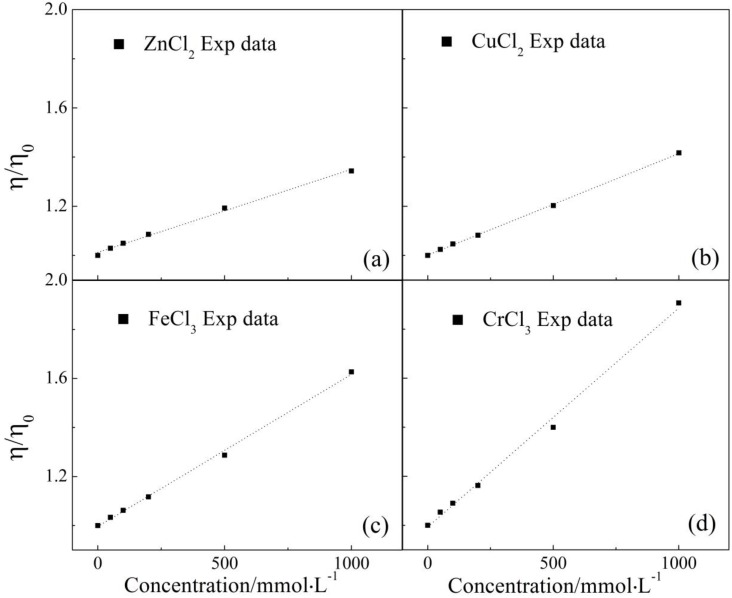
Specific viscosity of ZnCl_2_ (**a**), CuCl_2_ (**b**), FeCl_3_ (**c**) and CrCl_3_ (**d**) aqueous solutions at different concentrations. Short dashed lines are the linear fitting results corresponding to the experimental data.

In 1929, Jones and Dole [30] came up with the semi-empirical equation, Jones–Dole equation, to account for the relationship between viscosity and solution concentration. Especially when solute concentration is lower than 1 mol·L^−1^, the equation could be reduced as follows:

(6) $$\eta /\eta_{0}=1+B_{\text{vis}}C$$

where *η* and *η*_0_ are the viscosity of salt solution and pure water, respectively, m^2^·s^−1^. *C* is the solution concentration, mmol·L^−1^. *B*_vis_ is the viscosity coefficient, L·mmol^−1^, and used to describe the interaction between solute and solvent molecule. The Jones–Dole equation proves that the viscosity of less concentrated solution is dominated by solute–solvent interaction and local structure surrounding center metal ions. Here, specific viscosity and corresponding fitted lines of different solutions could be seen in Figure 3. For the four electrolytes, the specific viscosity increased with salt concentration in a linear fashion, which is supported by the acceptable goodness of fit (see Table 1). The linear fitting results are tabulated in Table 1.

Based on the Jones–Dole equation, the *B*_vis_ for the four solutes (see Table 1), consistently larger than zero which is the characteristic value for pure water, was the supportative evidence that the four metal ions can interact with the adjacent water molecules intensively to form ionic cluster, similar to those hydrated metal ions found by Extended X-ray Absorption Fine Structure (EXAFS) [31,32,33,34]. Even though the size of ionic cluster predicted here may be different from those of hydrated metal ions, the *B*_vis_ confirmed that the four metal ions are capable of increasing the association degree of adjacent water cluster to some extent.

Besides concentration, ionic species is another factor affecting solution viscosity as indicated by *B*_vis_ (see Table 1). Cr(III) had the largest *B*_vis_, the second was Fe(III) followed by Cu(II), and Zn(II) bore the smallest *B*_vis_. Thus, the ability in increasing association degree decreased in the order of Cr^3+^ > Fe^3+^ > Cu^2+^ > Zn^2+^. Furthermore, according to the Einstein equation [35], the size of the ionic cluster could be compared by using the specific viscosity of the four aqueous solutions with the same concentration. As a result, we found that Cr(III) was characterized by the largest effective radius, the second was Fe(III), and Cu(II) and Zn(II) had the third largest and the smallest effective radius, respectively. This order is identical to that of *B*_vis_, and those of *D* and *B*_NMR_ from NMR measurements, which proved that the four metal ions chosen here can enhance association degree of local water cluster with some regularities.

### 2.3. Raman Characterization

**Figure 4:**
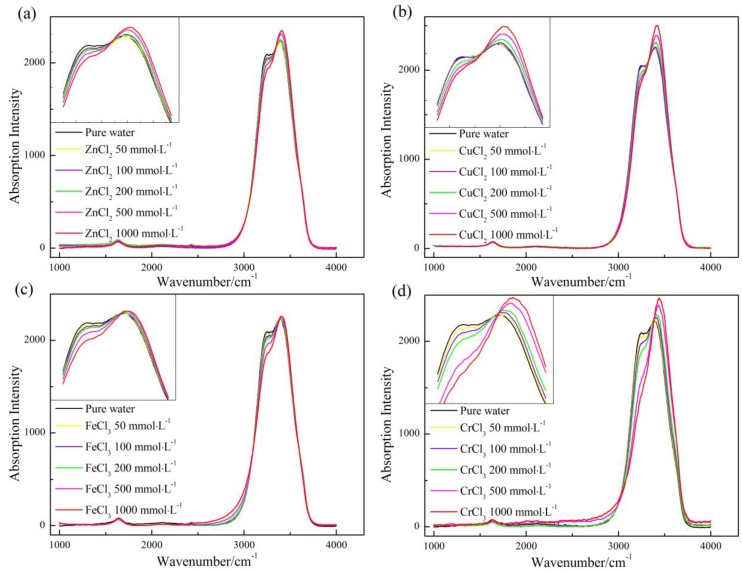
Comparison of Raman spectrum of aqueous solutions with different concentrations. (**a**) Raman spectrum of ZnCl_2_ solutions; (**b**) Raman spectrum of CuCl_2_ solutions; (**c**) Raman spectrum of FeCl_3_ solutions; (**d**) Raman spectrum of CrCl_3_ solutions. Insets: magnified OH stretching vibration peak ranging from 3100 to 3600 cm^−1^.

Mid-infrared Raman/IR spectroscopy of aqueous solution has been widely employed to probe into the water structure and the effect of solutes on water cluster in the field of solution chemistry [36,37,38]. The Raman spectrum of a variety of aqueous solutions with different concentrations were collected (see Figure 4).

The Raman spectrum of the four salt solutions were all characterized by a weak peak at ~1600 cm^−1^ and a strong peak (OH stretching vibration) ranging from 3000 to 3700 cm^−1^. However, the shape of OH stretching peak presented remarkable changes with solute concentrations regardless of metal chloride. By scrutinizing the insets of Figure 4, one can see an intersection point existing among the multiple spectrums, and the peak on the left-side of the point got weakened with salt concentration, while the peak on the right-side changed in an opposite manner. According to the working principle of Raman spectroscopy, the opposite changes in the peak intensity means that the amount of high association component becomes fewer and fewer upon solute addition, while the amount of low association component increases at the same time. It is weird to see the changing manner of water structure, when considering our NMR and viscosity results. The reason leading to the discrepancy between Raman, NMR and viscosity results was discussed in Section 3.

**Figure 5:**
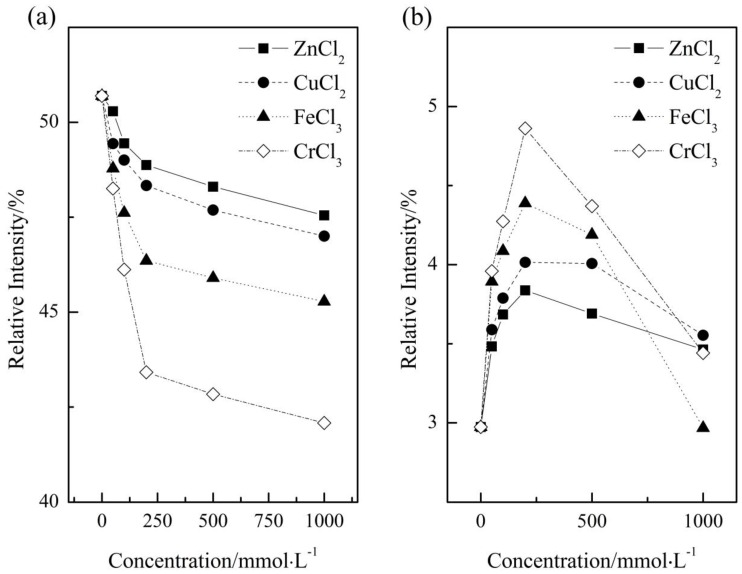
Changes in content of high association water (**a**) and low association water (**b**) with concentration. The left-most points in the two panels correspond to the relative content of pure water.

Deconvolution of OH stretching peak into sub-peaks is a powerful tool to probe into water structure, provided suitable fitting strategy is chosen [37,39]. Here, according to Carey and Korenowski [40], the Raman spectrum of different aqueous solutions were decomposed into five sub-peaks centered at 3051, 3233, 3393, 3511, and 3628 cm^−1^, respectively. It is widely accepted that, the higher the association degree is, the lower the vibrational energy is [37,39]. Thus, the two sub-peaks centered at 3051 and 3233 cm^−1^ corresponded to the species with high association degree, the sub-peak centered at the highest wavenumber corresponded to the low association water, and the other two peaks corresponded to the species with medium association degree. On the basis of which, the variations in content of two species with concentration were shown, quantitatively, in Figure 5.

According to the fitting results (see Table S1), the goodness of fit, *R*^2^, were all higher than 99.8%, which means that the fitting results are mathematically reasonable. From Figure 5, the content of high association water declined consistently with salt concentration, while the content of low association water increased rapidly before 200 mmol·L^−1^ and then decreased with salt concentration. The decrease in the amount of low association water, as pointed out by Pestova *et al.* [10] and O’Brien and Williams [12], is due mainly to the overlap of hydration layer with the continuous increase in metal ion concentration. However, the addition of the electrolytes promotes the decrease in association degree, which is in contrast to the regularities obtained from NMR and viscosity measurements and needs to be further discussed.

**Figure 6:**
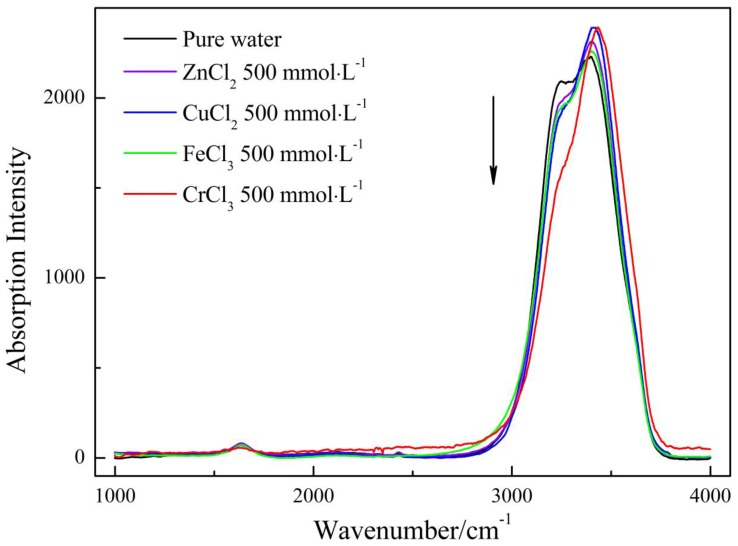
Comparison of Raman spectrum of pure water and four electrolyte solutions at 500 mmol·L^−1^.

Moreover, the impact on water structure is ion-specific as shown in Figure 5. Concretely, the high association water and low association water were characterized by the largest amplitude of variation upon CrCl_3_ addition, and FeCl_3_ and CuCl_2_ were in the second and third place, respectively, and ZnCl_2_ had the minimum effect on the amount of the two species. To compare the influence induced by the four electrolytes, we plotted the normalized Raman spectrum of pure water and four aqueous solutions with the same concentrations (500 mmol·L^−1^) in Figure 6. From Figure 6, one can see a main peak and a shoulder peak centered at the lower wavenumber for the five solutions, among which, pure water had the strongest shoulder peak, second place was acquired by ZnCl_2_, CuCl_2_ and FeCl_3_ had similar peak intensity, and CrCl_3_ was characterized by the weakest shoulder peak. This relative position is almost identical to those obtained from other experimental measurements occupied in this article.

### 2.4. Molecular Dynamics Simulations

**Figure 7:**
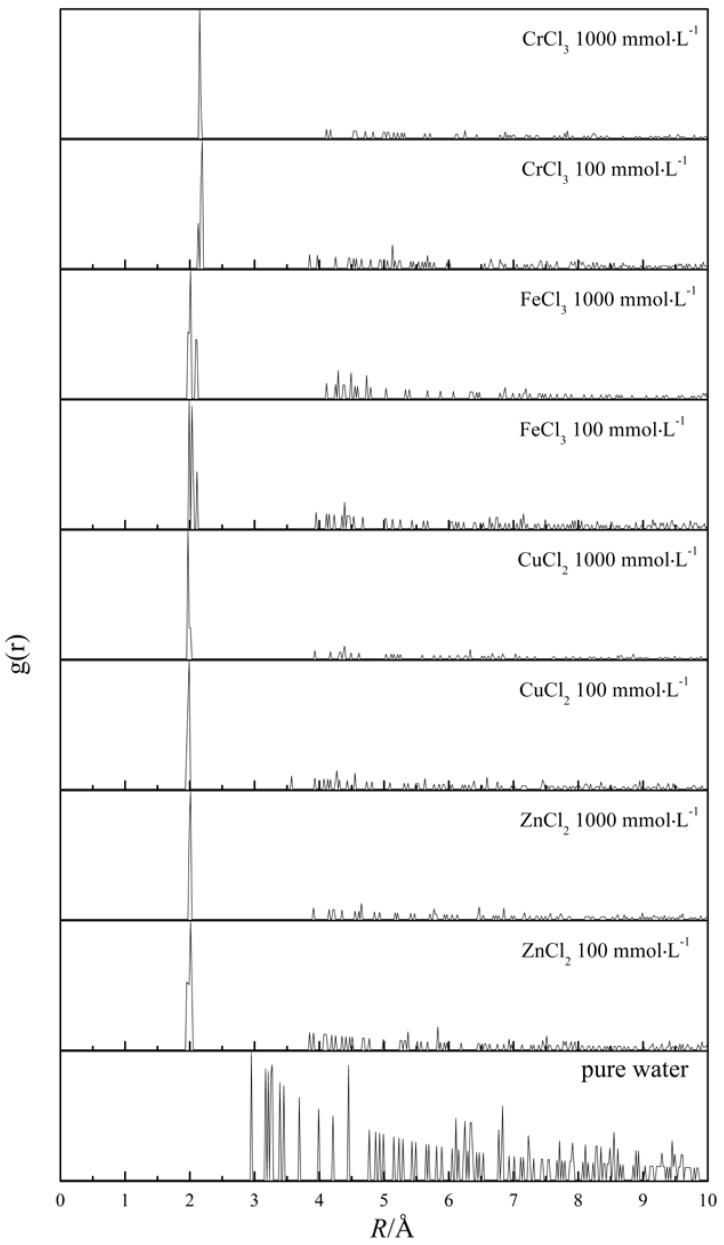
RDF of pure water and four electrolyte solutions at two concentrations.

In addition to the experimental methods, computer simulations is another alternative to study the effect of metal ions on water structure from the perspective of microscopic particle interactions. Here, molecular dynamics simulations were used to represent the spatial arrangement of water molecules around the center metal ions. Firstly, the distance of center water molecule and the nearest water molecule in the first coordination layer (O–O) was calculated to be 2.9–3.0 Å, which was similar to another MD simulation result (2.80 Å) [41], and even closer to an spectral result (2.976 Å) [42]. This characteristic value was also manifested by the first peak of pure water RDF in Figure 7. Furthermore, except for specific position, e.g., ~4.5 Å, the RDF of pure water decreased gradually with distance, which means that the disorder degree of water structure increases with distance in a gentle manner.

As for electrolyte aqueous solutions, the RDF was obtained by calculating the appearance probability of oxygen atoms surrounding center metal ions. From Figure 7, one could observe that the first hydration layer was located ~2.0 Å away from center metal ions, which was identical to the corresponding value reported in the literature [31,32,33,34]. Nevertheless, the RDF of the four aqueous solutions fell to zero rapidly and then presented weak peaks at further distances. In addition, the RDF of aqueous solutions at low concentration (100 mmol·L^−1^) had stronger peaks at 4–8 Å than those at high concentration (1000 mmol·L^−1^). The stark contrast in RDF between pure water and electrolyte aqueous solution proved that the increased content of low association water observed by Raman spectrum might be the result of the increased disorder degree of water structure at further distance induced by solute addition.

Furthermore, for the solutions at the same concentration, an Fe-containing system was characterized by the remarkable long-range order as indicated by the strongest RDF peak at 4–8 Å. Comparatively, the other three metal ions could further break the long-range ordered water structure to a similar extent. Namely, there is no apparent ion-dependent regularity, which still remains to be answered.

## 3. Discussion

Several experimental methods together with MD simulations were adopted by us to characterize the influence of metal ions on water structure. Firstly, the four metal ions (Cr^3+^, Fe^3+^, Cu^2+^, Zn^2+^) studied here have been classified as kosmotropes by Collins [43], as their strong interactions with adjacent water molecules than water with itself. Thus, the dissolved metal ions can pose a restriction on translational motion of a number of water molecules, and further promote their association. Accordingly, this confinement on adjacent water molecule have been confirmed by NMR coefficient (*B*_NMR_) and diffusion coefficient (*D*) from NMR, viscosity coefficient (*B*_vis_) from viscometer. However, our Raman spectrum suggested that ion addition would decrease the content of high association water. The stark contrast among the experimental results presented above may just come from the difference in the working principle of these detection methods. NMR is the technique that can emit radio frequency radiation to detect sample structure with the characteristic time-scale of 10^−9^–10^−6^ s [44]. By comparison, Raman spectroscopy is capable of catching molecular vibration/rotation information on shorter time-scales (10^−12^–10^−9^ s) by producing electromagnetic waves [44,45]. In addition, ultrafast laser studies have confirmed that the dynamics of the HB network is pretty fast in pure water; specifically, the spectral diffusion time for HDO/D_2_O system and the vibrational lifetime in pure H_2_O are less than 200 fs [46], and ~200 fs [47], respectively. Furthermore, Cowan *et al.* [48] found the timescale of energy redistribution within the HB network was less than 50 fs by using 2D IR spectroscopy. Thus, Raman spectroscopy would be more sensitive to all shorter-lived water species with different association degree relative to NMR. Additionally, as for viscosity measurement, specific viscosity, *η*/*η*_0_ is the characterization of ionic-centered water cluster according to the Einstein equation [35], which is similar to the results obtained from NMR. Thus, viscosity, NMR and Raman spectroscopy are all able to present structural features of solution system but attach their importance on different aspects of solution system.

Very recently, a THz TDS study concluded that the saccharides having strong hydration capacity can destruct the HB network of bulk water [49]. Additionally, a dielectric relaxation study also found the opposite effects of ions on water structure, namely, a decelerating effect within the hydration shell and breaking effect beyond the hydration shell [20]. Thus, those findings have pointed out the destructing effect on water structure, which is also observed in this article by ingenious deconvolution of Raman spectrum. Furthermore, as shown in Figure 7, the distant RDF peaks, e.g., 4–8 Å, got weaker with salt concentration increasing. Thus, the destructing effect comes mainly from the ion-related influence on long-range water structure. By combining the experimental and simulation results, it is confirmed that the dissolved metal ions studied here (Cr^3+^, Fe^3+^, Cu^2+^, Zn^2+^) can elevate the association degree of adjacent water molecules, but break the ordered water cluster far away from center ion simultaneously, just at the presence of collective hydrogen bond dynamics existing among water molecules [50]. The point summarized here can also shed light on the opposite effect of dissolved cations on dielectric spectra between the hydration shell and the outer shell [20].

Additionally, the water structural changes were also found to be dependent on the nature of metal ions. Specifically, the ability in alternating water structure decreased in the order of Cr^3+^ > Fe^3+^ > Cu^2+^ > Zn^2+^ as consistently manifested by NMR, viscometer and Raman measurements. In 2012, a statistical mechanics study found the cation-induced changes in molecular orientation and non-linear polarization effects could persist up to ~3–4 hydration layers for low charge density ions, and ~7–9 layers for high charge density ions [19]. Recently, we also found the charge density is the key to the influence of cations on water structure [51]. Thus, given the experimental results here, it is very likely that surface charge density plays the key role in affecting the association degree of local water cluster, as Cr^3+^ and Fe^3+^ bear more charges and smaller ionic radii, while Cu^2+^ and Zn^2+^ have fewer charges and larger ionic radii [52].

## 4. Materials and Methods

### 4.1. Materials

ZnCl_2_ (guaranteed reagent) was supplied by Kermel Chemical Reagent Company (Tianjin, China). CuCl_2_·2H_2_O (guaranteed reagent) was purchased from Sangon Biotech Co., Ltd. (Shanghai, China). FeCl_3_·6H_2_O and CrCl_3_·6H_2_O were of analytical grade and were the products of Aladdin Industrial Corporation (Shanghai, China). Other chemicals were of analytical grade and used without further purification. The solutions used in NMR, viscosity and Raman characterizations were obtained by dissolving solutes into ultrapure water (18.2 MΩ·cm).

### 4.2. NMR Measurements

A series of ZnCl_2_, CuCl_2_, FeCl_3_, CrCl_3_ aqueous solutions and pure water were put into NMR tubes to perform NMR experiments on a Bruker AV-600 MHz NMR spectrometer equipped with a BBO probe (Bruker, Karlsruhe, Germany). ^17^O NMR spin-lattice relaxation time (*T*_1_) of solution samples were measured using the π-τ-π/2 pulse sequence with the characteristic pulse width of π/2, 13.8 μs. *T*_1_ were obtained by fitting signal intensity at 14 relaxation time ranging from 0.01 to 100 ms to the following equation:

(7) $$S_{\tau }=S_{0}\left(1-e^{-\tau /T_{1}}\right)$$

where *τ* is the relaxation time, ms, and *S*_τ_ and *S*_0_ are the signal intensity at relaxation time zero and *τ*, respectively. Importantly, to prevent incorrect *T*_1_ from being returned because of the exchange interaction existing between electron spin and proton spin [53], the top concentration for Cr, Cu, Zn-containing system was set to be 3 mmol·L^−1^. Because of ferromagnetism, the concentration for Fe-containing system was further limited to be less than 1 mmol·L^−1^.

The aqueous solutions at two concentrations (1 and 3 mmol·L^−1^ for ZnCl_2_, CuCl_2_, CrCl_3_, 0.25 and 1 for FeCl_3_) and pure water/deuterated water were put into NMR tubes to perform 2D Diffusion-Ordered Spectroscopy (DOSY) ^1^H NMR experiments. Constant temperature (297.9 K) DOSY data were acquired using an Smoothed-Square (SMSQ)-shaped 10.100 pulse sequence with 2 ms gradient pulse duration, 20 s time interval between neighbor pulses. Complete signal attenuation was achieved by exponentially increasing the gradient strength from 2%–98% with 8 scans each. The spin-echo attenuation was related to the pulse sequence parameters and the diffusion coefficient, *D*, by the following equation:

(8) $$\text{ln}\frac{I}{I_{0}}=-(2\pi \gamma g_{z}{\delta )}^{2}D\left(\Delta -\frac{\delta }{3}\right){\times 10}^{4}$$

where *I* and *I*_0_ are the intensity of signal and original pulse, respectively. *D* is the diffusion coefficient, m^2^·s^−1^, *γ* is the gyromagnetic ratio of protons, MHz·T^−1^, *g*_z_ is the gradient amplitude, *δ* is the gradient duration, ms, and *Δ* is the diffusion time.

### 4.3. Viscosity Measurements

The aqueous solutions with the different concentrations, 50, 100, 200, 500, and 1000 mmol·L^−1^ for the four electrolytes, were prepared in advance. The density of aqueous solutions and ultrapure water were measured by pycnometer method. The time for solution samples to descend from scale A (upper limit) to scale B (lower limit) was obtained by using Ubbelohde viscometer. The specific viscosity was calculated according to the following equation:

(9) $$T=\frac{\eta }{\eta_{0}}=\frac{\rho \times t}{\rho_{0}{\times t}_{0}}$$

where *T* is the specific viscosity, *η* and *η*_0_ are the viscosity of aqueous solution and ultrapure water, respectively, m^2^·s^−1^, *ρ* and *ρ*_0_ are the density of aqueous solution and ultrapure water, respectively, kg·m^−3^, *t* and *t*_0_ are the residence time of aqueous solution and ultrapure water between scale A and B, respectively, s. The measurements of density and viscosity were all conducted under water bath of 298.15 K. All assays were carried out in triplicate and only mean values were presented in Section 2.2.

### 4.4. Raman Characterization

The Raman spectra of aqueous solutions with concentrations identical to those in viscosity measurements, were all recorded at 300.15 K by a confocal microscope Raman spectrometer (Renishaw Invia Reflex, Wotton-under-Edge, UK) equipped with a 532 nm YAG laser. The laser power was 7.05 mW. The Raman spectra of aqueous solutions were collected in the region between 1000 and 4000 cm^−1^ with spectral resolution of approximately 1 cm^−1^. The laser beam was focused on the solution sample at a characteristic depth of 1993 μm from the outer surface of quartz cuvette. After smoothing, baseline correction, Raman spectra were normalized based on the OH stretching vibration peak intensity in OriginPro 8 (OriginLab Corporation, Northampton, MA, USA). The broad OH stretching vibration peak was deconvoluted and fitted by using PeakFit v4.12 (SeaSolve Software Inc., Framingham, MA, USA).

### 4.5. Molecular Dynamics Simulations

All the structures were optimized by using Molecular Dynamic (MD) simulations as implemented in the Materials Studio 7.0 (Accelrys, San Diego, CA, USA). Firstly, metal ions (Cr^3+^, Fe^3+^, Cu^2+^, Zn^2+^) and water molecule were constructed in the Materials Visualizer module. Then, two cubic simulation boxes corresponding to 100 mmol·L^−1^ (containing 5 metal ions and 275 water molecules) and 1000 mmol·L^−1^ (containing 5 metal ions and 2775 water molecules) aqueous solutions were constructed in Amorphous Cell module for each metal. Meanwhile, Condensed-Phase Optimized Molecular Potentials for Atomistic Simulation Studies (COMPASS) force field parameters were assigned to every particles. Furthermore, MD simulations were conducted for each canonical ensemble (NVT system) characterized by 1 g·cm^−3^ with time step of 1 fs, and dynamics time of 5 ps at 298 K. MD optimized structure for Fe-containing aqueous solutions can be seen in Figure S1. On this basis, RDFs for each optimized system were obtained by using radial distribution analysis in the Forcite module with a specific cutoff of 10.0 Å. Finally, the RDF data were normalized and plotted in OriginPro 8 (OriginLab Corporation).

## 5. Conclusions

This study has shown that Cr^3+^, Fe^3+^, Cu^2+^ and Zn^2+^ have remarkable effects on water structure. The combination of NMR, viscosity, Raman and MD simulations allowed us to get a deep insight into the effect of different metal ions and their concentration on short-/long-range water structure. NMR and viscosity data pointed out that all of the four metal ions studied here can improve the association degree of adjacent water clusters and form ionic clusters with neighbor water molecules. The ability in increasing association degree decreased in the order of Cr^3+^ > Fe^3+^ > Cu^2+^ > Zn^2+^. Meanwhile, the analysis of Raman spectrum showed that the content of high association water declined consistently, while the content of low association water increased rapidly and then decreased with salt concentration. Furthermore, with the help of MD simulations, it is found that dissolved metal ions can increase the association degree of adjacent water molecules, but also break the ordered water structure beyond the adjacent area.

The experimental characterization/MD simulations presented herein proved to be extremely useful for studying the changes in water structure with collective hydrogen bond dynamics induced by metal ions. Meanwhile, the regularities obtained in this article may be helpful to explain the determinant effects of dissolved metal ion on the chemical and biological properties of ionic solution.

## Figures and Tables

**Table 1:** Fitting results of specific viscosity *versus* concentration for four electrolyte aqueous solutions. *B*_vis_: viscosity coefficient; *T*: intercept of Equation (6).

System	*B*_vis_ (L·mmol^−1^)	*T*	*R*^2^
ZnCl_2_	3.3790 × 10^−4^	1.0125	0.9946
CuCl_2_	4.1319 × 10^−4^	1.0014	0.9994
FeCl_3_	6.2041 × 10^−4^	0.9964	0.9974
CrCl_3_	8.9294 × 10^−4^	0.9935	0.9946

## Supplementary Materials

Supplementary materials can be found at http://www.mdpi.com/1422-0067/17/5/602/s1.
