# Supplementary material for: The Opposite Effect of Metal Ions on Short-/Long-Range Water Structure: A Multiple Characterization Study
Source: Int J Mol Sci. 2016 Apr 25;17(5):602. doi: 10.3390/ijms17050602 (PMC4881438; doi:10.3390/ijms17050602)
Supplement: Supplementary file 1 [file ijms-17-00602-s001.pdf]

Supplementary Materials: The Opposite Effect of Metal Ions on Short-/Long-Range Water Structure: A Multiple Characterization Study

Kai Ma and Lin Zhao

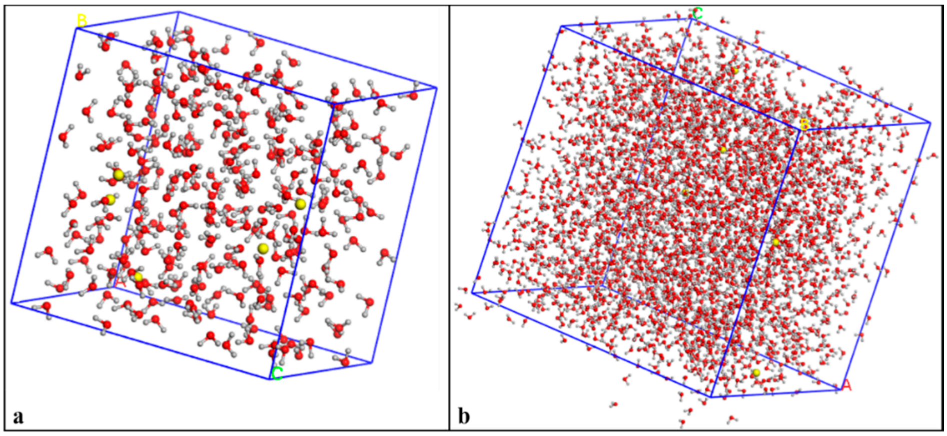

Figure S1. MD optimized structure for 1000 (**a**) and 100 (**b**) mmol·L^{-1} Fe^{3+} aqueous solution. Yellow particles represent to Fe^{3+}. Red particles represent to oxygen atoms of water molecules.

Table S1. Fitting results by deconvoluting Raman OH stretching band into five sub-bands.

System	Concentration/ mmol·L^{-1}	Relative Intensity/%					R^2 *
		3051 cm^{-1}	3233 cm^{-1}	3393 cm^{-1}	3511 cm^{-1}	3628 cm^{-1}	
$ZnCl_2$	50	8.8812	41.4072	25.3779	20.8504	3.4833	0.9981
	100	6.4613	42.9832	25.9663	20.9044	3.6848	0.9993
	200	8.9736	39.9017	26.1752	21.1119	3.8376	0.9980
	500	8.2343	40.0695	27.1025	20.9028	3.6909	0.9989
	1000	9.3044	38.2455	28.9551	20.0313	3.4637	0.9992
$CuCl_2$	50	6.6592	42.7803	25.3087	21.6625	3.5893	0.9993
	100	6.9480	42.0576	25.4306	21.7752	3.7886	0.9992
	200	7.4653	40.8697	25.7141	21.9359	4.0150	0.9990
	500	7.7003	39.9895	26.8416	21.4616	4.0070	0.9993
	1000	10.9677	36.0339	25.0684	24.3763	3.5537	0.9981
$FeCl_3$	50	6.0204	42.7654	26.4556	20.8650	3.8936	0.9996
	100	7.2259	40.3906	25.8157	22.4812	4.0866	0.9995
	200	8.2179	38.1374	26.3740	22.8821	4.3886	0.9995
	500	9.2557	36.6473	26.7883	23.1194	4.1893	0.9994
	1000	14.1452	31.1353	30.4406	21.3103	2.9686	0.9993
$CrCl_3$	50	6.7215	41.5348	25.7353	22.0497	3.9587	0.9994
	100	7.0103	39.1107	26.5389	23.0658	4.2743	0.9996
	200	7.7885	35.6284	27.0652	24.6571	4.8608	0.9996
	500	13.1757	29.6650	26.8882	25.9008	4.3703	0.9994
	1000	16.9724	25.1086	27.4898	26.9879	3.4413	0.9991

*: Refers to goodness of fit.
